# Unveiling Tuberous Sclerosis in a Diagnosed Patient With Systemic Lupus Erythematosus: A Rare Coincidence

**DOI:** 10.7759/cureus.62133

**Published:** 2024-06-11

**Authors:** Karthiga Selvaratnam, Pakkiyaretnam Mayurathan

**Affiliations:** 1 University Medical Unit, Teaching Hospital Batticaloa, Batticaloa, LKA; 2 Department of Clinical Sciences, Faculty of Health-Care Sciences, Eastern University of Sri Lanka, Batticaloa, LKA

**Keywords:** angiomyolipomas, angiofibroma, tuberous sclerosis complex, mtor signalling, systemic lupus erythematosus

## Abstract

Systemic lupus erythematosus (SLE) is a chronic autoimmune disease presenting a variable clinical course ranging from mild to severe multiorgan dysfunction. While the exact etiology of SLE remains elusive, genetic and environmental factors are known to play crucial roles in its pathogenesis. Similarly, tuberous sclerosis complex (TSC) is a multisystem autosomal dominant genetic condition that manifests as benign hamartomatous proliferation in various organs. We present the case of a 46-year-old woman diagnosed with SLE who exhibited clinical features of TSC two decades after the initial diagnosis of SLE. The definitive diagnosis of TSC was made based on major clinical criteria, including facial angiofibroma and bilateral renal angiomyolipomas. As the patient remained asymptomatic without neurological complications, specific treatment for TSC was not initiated. The coexistence of SLE and TSC is exceedingly rare and has been scarcely reported in medical literature.

## Introduction

Systemic lupus erythematosus (SLE) is a chronic inflammatory multisystem disorder of autoimmune origin, characterized by the production of autoantibodies against nuclear antigens [1]. It predominantly affects women and typically manifests after puberty, presenting with diverse clinical features affecting various organs. The multifactorial pathogenesis of SLE involves genetic, epigenetic, and environmental factors, with ethnicity and geographical location influencing disease prevalence and severity [1].

Recent research has highlighted the crucial role of the mTOR signaling pathway in SLE pathogenesis, with mTOR inhibitors such as rapamycin showing promise as targeted treatments for SLE [2]. Dysregulation of the mTOR pathway is implicated in several diseases, including autoimmune disorders, cancer, neurological conditions, and metabolic disorders [3].

Tuberous sclerosis complex (TSC) is another condition associated with aberrant mTOR signaling, resulting from mutations in the TSC1 or TSC2 suppressor genes [4]. Patients with TSC might have a higher risk of developing SLE because both share a common mTOR signaling pathway and trigger autoimmunity [5].

TSC manifests as benign hamartomatous proliferation in various organs, including the brain, kidneys, lungs, skin, heart, and eyes. Multifocal micronodular pneumocyte hyperplasia (MMPH) is a rare lung manifestation of TSC, characterized by the proliferation of type II pneumocytes due to hyperphosphorylated mTOR-related proteins. There is no specific treatment for MMPH [6].

## Case presentation

Our patient is a 46-year-old female with a history of systemic lupus erythematosus (SLE), which was diagnosed at the age of 26 based on new EULAR/ACR classification criteria (clinical criteria: oral ulcers, arthritis, hemolytic anemia, and thrombocytopenia) and serological evidence of positive antinuclear antibodies and double-stranded DNA. She never had renal or central nervous system involvement. She was treated with prednisolone and azathioprine to manage her SLE during remission. She was only on azathioprine at the time of admission. This time she presented with a low-grade fever and cough with a sputum of one week duration. There was no history of hemoptysis, evening pyrexia, or contact history of tuberculosis. She denied pleuritic-type chest pain or exertional shortness of breath. There was no history of hematuria or dysuria. Urine output was adequate. She didn't have any altered bowel habits. She never had seizures, confusion, or any other neurological features. There was no family history of TSC.

**Figure 1:**
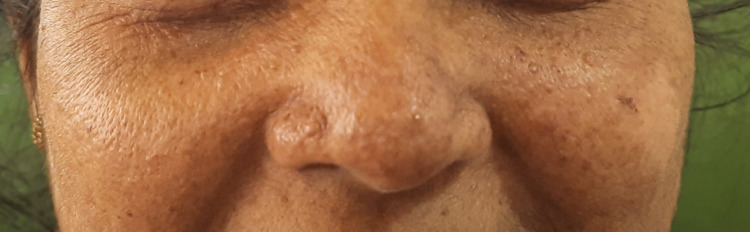
Facial angiofibroma

On examination, she was mildly pale but not icteric. Further examination revealed multiple erythematous papules on her face, consistent with facial angiofibroma (Figure 1). There was no clubbing or any nail abnormalities. All her other system examinations are unremarkable. 

Laboratory findings are as follows (Table 1 ). The blood picture showed mild microcytic hypochromic anemia due to iron deficiency. The liver function test, urine full report, and chest X-ray were unremarkable. There was no proteinuria or microscopic hematuria.

**Table 1:** Laboratory values

Lab tests	Result	Normal range
White blood count (WBC)	8.4x10^3^/uL	4.00 to 11.00 x10^3^/uL
Hemoglobin (Hb)	10.3 g/dl	11 to 15 g/dl
Platelet	156x10^3^/uL	150 to 450x10^3^/uL
Serum creatinine	1.01 mg/dl	0.5 to 1.1 mg/dl
Serum sodium	137 mmol/L	136 to 145 mmol/L
Serum potassium	3.6 mmol/L	3.5 to 5.1 mmol/L
Erythrocyte sediment rate (ESR)	20 mm/h	< 20 mm/h
C reactive protein (CRP)	50 mg/dl	0 to 5 mg/dl

She was managed as a lower respiratory tract infection with oral co-amoxiclav 625 mg three times per day for five days and oral hematinics for mild iron deficiency anemia. A dermatology opinion was sought for a facial rash, which was confirmed to be facial angiofibroma, raising the possibility of TSC.

Ultrasound abdomen revealed multiple well-defined hypoechoic lesions favoring bilateral multiple renal angiomyolipoma (AML) with no features of chronic kidney disease and normal liver architecture. The identification of bilateral AML and facial angiofibroma prompted the diagnosis of TSC based on clinical criteria. Genetic tests were not done due to their unavailability.

**Figure 2:**
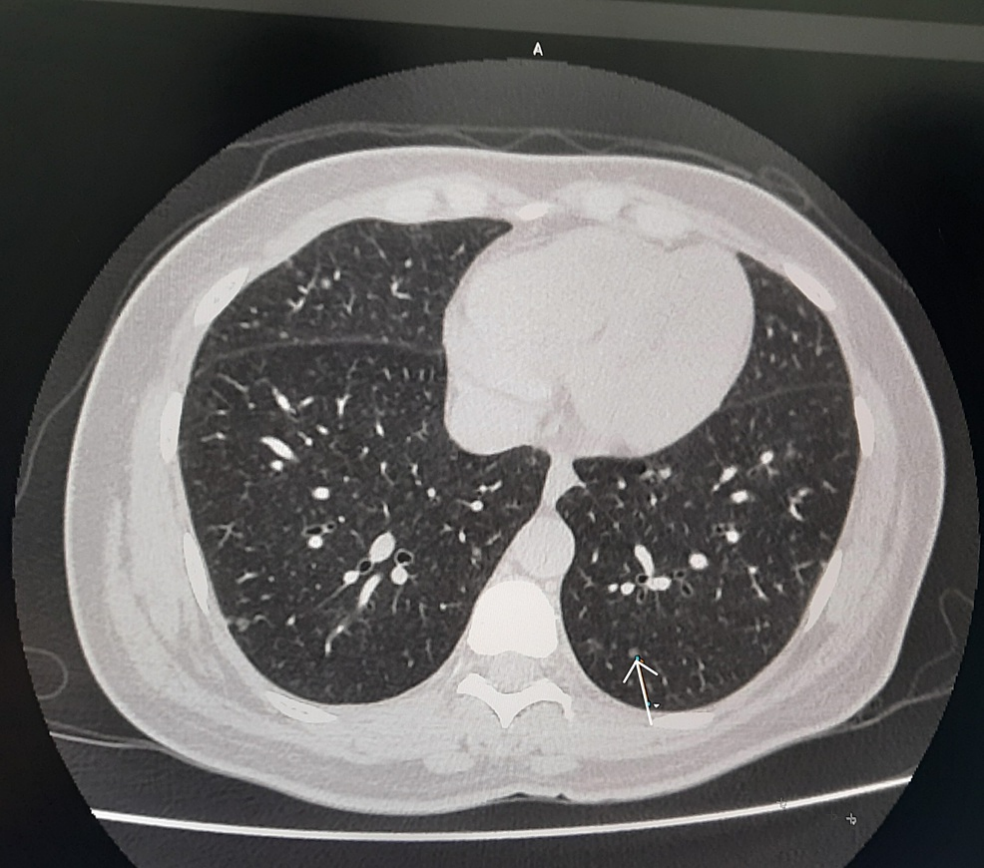
High-resolution computed tomography (HRCT) chest showing multiple 3-5 mm-size pulmonary nodules seen in all lobes of both lungs favor multifocal micronodular pneumocyte hyperplasia (MMPH) with no features of lymphomyomatosis

High-resolution computed tomography (HRCT) of the chest revealed multiple 3-5 mm sized pulmonary nodules seen in all lobes of both lungs, favoring multifocal micronodular pneumocyte hyperplasia (MMPH) with no features of lymphangiomyomatosis (Figure 2).

Her echocardiogram was normal and did not show any evidence of rhabdomyoma. There was no retinal hamartoma on the ophthalmic examination. There were no sclerotic bone lesions or any other dermatological manifestations. The diagnosis was made based on two major criteria.

Specific treatment for TSC was not initiated as this patient was asymptomatic, without neurological manifestations, and clinically stable. She was discharged with oral antibiotics and haematinics with the plan to continue routine medications such as Azathioprine. She was instructed to continue the rheumatology as well as the medical clinic follow-up. Annual follow-up scans were arranged on discharge.

## Discussion

The coexistence of tuberous sclerosis complex (TSC) and systemic lupus erythematosus (SLE) is exceedingly rare and poses diagnostic and management challenges. While both conditions involve dysregulation of the mTOR signaling pathway, their clinical presentations and disease courses vary significantly.

In our case, the patient exhibited clinical signs of TSC two decades after the diagnosis of SLE, emphasizing the need for vigilance in monitoring patients with autoimmune diseases for potential comorbidities. The diagnosis of TSC was based on fulfilling two major clinical criteria (Table 2) [7], highlighting the importance of comprehensive evaluation and consideration of rare disease entities. There have been a few cases reported regarding the coincidence of both diseases, similar to the case we are discussing [5,8].

**Table 2:** Diagnostic criteria for TSC: A definitive TSC requires two major criteria or one major and two or more minor criteria. A possible TSC requires either one major criterion or two or more minor criteria Table credits: Corresponding author

Major Criteria	Minor Criteria
Hypermelanotic macules (> 3, at least 5mm diameter	Confetti skin lesions (1 to 2 mm hypomelanotic macules)
Angiofibromas (>3) or fibrous cephalic plaque	Dental enamel pits (>3)
Ungual fibromas (>2)	Intraoral fibromas (>2)
Shagreen patch	Retinal achromic patch
Multiple retinal harmatomas	Multiple renal cysts
Multiple cortical tubers and /or radial migration line	Nonrenal harmatoma
Subependymal nodules (>2)	Sclerotic bone lesions
Subependymal giant cell astrocytoma	
Cardiac rhabdomyoma	
Lymphangiomyomatosis	
Angiomyolipomas (>2)	

TSC is a neurocutaneous disorder that affects multiple organs in the body, mainly the skin, kidney, brain, and lung. Although the classic triad of seizures, intellectual disability, and facial angiofibroma is a hallmark of TSC, only one-third of the patients exhibit these traits [6]. In our case, she never had seizures or neurological symptoms. However, since childhood, she has had a rash of erythematous nodules on her nose and cheek, which recently became more pronounced and required medical attention.

Renal angiomyolipoma (AML) is a common renal manifestation of TSC, comprising 49-60% of TSC patients overall. Most of the patients are asymptomatic. Patients with the following features need treatment: size greater than 6 cm, number of angiomyolipomas, rate of growth greater than 2.5 mm per year, malignant transformation, bleeding into the tumor, or features of chronic kidney disease. Patients without the above-mentioned features, similar to ours, need yearly surveillance with an ultrasound, CT, or MRI scan [6]. Treatment options for TSC-related AML include surveillance, embolization, surgical excision, or mTOR inhibitors in select cases [6,9].

Multifocal micronodular pneumocyte hyperplasia (MMPH) is less common than lymphangiomyomatosis (LAM) in TSC. There is no specific treatment for MMPH because of its nonprogressive, benign nature. In our case, HRCT chest findings confirmed the presence of MMPH without any features of LAM.

In our case, azathioprine maintains stability in SLE without any renal involvement. Continuous surveillance and follow-up are necessary for TSC with renal AML and MMPH.

## Conclusions

This case report highlights the rare coexistence of TSC and SLE. The presence of TSC was identified 20 years after the initial diagnosis of SLE, illustrating the importance of vigilance for potential overlapping syndromes in patients with autoimmune diseases. This case underscores the necessity for continuous monitoring and multidisciplinary management approaches. Further research is needed to elucidate the underlying mechanisms linking these conditions and optimize therapeutic strategies for affected patients.
